# Optimized LOAM Using Ground Plane Constraints and SegMatch-Based Loop Detection

**DOI:** 10.3390/s19245419

**Published:** 2019-12-09

**Authors:** Xiao Liu, Lei Zhang, Shengran Qin, Daji Tian, Shihan Ouyang, Chu Chen

**Affiliations:** 1 Faculty of Robot Science and Engineering, Northeastern University, Shenyang 110016, China; chenchu@siasun.com; 2 Shenyang SIASUN Robot & Automation Co., LTD., Shenyang 110168, China; zhanglei@siasun.com (L.Z.); qinshengran@siasun.com (S.Q.); tiandaji@gmail.com (D.T.); ouyangshihan@siasun.com (S.O.); 3 School of Information Science and Engineering, Shenyang University of Technology, Shenyang 110870, China

**Keywords:** SLAM, lidar, ground plane constraints, SegMatch, loop detection

## Abstract

Reducing the cumulative error in the process of simultaneous localization and mapping (SLAM) has always been a hot issue. In this paper, in order to improve the localization and mapping accuracy of ground vehicles, we proposed a novel optimized lidar odometry and mapping method using ground plane constraints and SegMatch-based loop detection. We only used the lidar point cloud to estimate the pose between consecutive frames, without any other sensors, such as Global Positioning System (GPS) and Inertial Measurement Unit (IMU). Firstly, the ground plane constraints were used to reduce matching errors. Then, based on more accurate lidar odometry obtained from lidar odometry and mapping (LOAM), SegMatch completed segmentation matching and loop detection to optimize the global pose. The neighborhood search was also used to accomplish the loop detection task in case of failure. Finally, the proposed method was evaluated and compared with the existing 3D lidar SLAM methods. Experiment results showed that the proposed method could realize low drift localization and dense 3D point cloud map construction.

## 1. Introduction

Over the last couple of decades, the application field of simultaneous localization and mapping (SLAM) has been paid more and more attention, especially in an intelligent vehicle. Compared with the visual sensor, the laser sensor has the advantages of high measurement accuracy, strong anti-interference ability, and wide sensing range, so the laser-based SLAM has higher positioning accuracy and better robustness. In the case of only using the lidar sensor, the pose change can only be calculated by matching between consecutive frames. In order to meet the real-time requirements, the pose estimation error obtained by the matching between frames and frames gradually increases when time changes, which is a typical problem in SLAM. Researchers have proposed loop detection algorithms to optimize global maps, thereby reducing drift errors. Unfortunately, the laser point cloud has only position information and lacks color information, so the environmental information features are less, which brings greater challenges to the laser SLAM. In addition, existing solutions for 3D closed-loop detection are computationally demanding.

In the existing 3D lidar-SLAM algorithm, lidar odometry and mapping (LOAM) [1] has been leading the way for the performance on the KITTI dataset, but the LOAM algorithm has only lidar odometry and no loop closure detection. This causes the drift error to increase over time. SegMatch [2] is a recognition algorithm that requires real-time odometry and does not work well when only using a lidar sensor.

Motivated by the discussion above, we proposed optimized lidar odometry and mapping method using ground plane constraints and SegMatch-based loop detection, which not only achieved robust pose estimation but also optimized global poses when detecting loop-closure. The main contributions of this paper were as follows:

1. We proposed optimized lidar odometry and mapping method. Ground plane constraints based on random sample consensus (RANSAC) [3] were added to reduce the matching errors. At the same time, SegMatch could perform loop detection efficiently so that the global pose could be optimized.
2. In order to verify our proposed solutions, extensive experiments were carried out in a variety of environments. Experiments showed that our method was suitable for completing inspection tasks and could also work well in the long-distance and large-scale outdoor environment.

The rest of the paper is organized as follows: Section 2 describes the related work about various point cloud registration and loop detection algorithms; Section 3 describes the proposed method in detail; Section 4 presents an experiment and analysis in different environment; and in the last section, the conclusions and expectations for future work are presented.

## 2. Related Work

The lidar-based SLAM has been the cornerstone of mobile robot mapping and navigation research for the past 20 years. Compared with visual sensors, lidar can provide more stable and accurate information and is less subject to external interference. Therefore, the laser can provide a more reliable solution for SLAM. The main work of lidar-based SLAM is frame matching, which is used to predict the position transformation between two adjacent frames. Typical point cloud registration methods are mainly iterative closest point (ICP) [4] and normal distribution transformation (NDT) [5]. When the number of point clouds is large, ICP will waste a lot of computing time. When NDT’s grid is set to be large, the matching accuracy is poor. Featured-based matching methods are more and more popular. [6] and [7] presented a key point selection algorithm, which calculated point curvature. [8] proposed a plane-based registration algorithm, but it could only be applied to indoor environments with many planes, and environments with fewer outdoor plane features limit such methods.

A low-drift and real-time lidar odometry and mapping (LOAM) method was proposed in [1] and [9]. The features are extracted by calculating the roughness of each point. The low roughness is the planar feature, and the roughness is the edge feature. Considering the real-time nature of the system, a combination of high-frequency coarse lidar odometry estimation and low-frequency accurate motion estimation is used. LOAM’s resulting accuracy is the best-achieved method that only uses lidar to estimate pose on the KITTI odometry benchmark site [10]. However, LOAM has no loop detection, and the accumulated error cannot be corrected.

Techniques for loop detection in 3D data can be broadly classified into two main categories. The one is local keypoint detection and matching, and the other one is global descriptor matching. The first category typically detects significant key points in the point cloud, calculates signatures for these keypoint locations, builds bag-of-words (BoWs), and finally matches them in different scans [11]. There are also many popular methods proposed, such as intrinsic shape signatures (ISSs) [12], Harris3D [13], Sift 3D [14], NARF [15], as well as many descriptors, such as spin images [16] and SHOT [17]. However, the detection of high repeatable key points remains a big challenge. For the lack of high repeatability issues, global descriptors, such as point feature histograms (PFH) [18] and viewpoint feature histograms (VFH) [19], have been proposed for using valuable techniques to extract features from point clouds. Recently, researchers tend to apply convolutional neural networks (CNN) to learn feature descriptors and to match their metrics in a uniform manner [20,21]. However, the limitation of using the deep learning method is that a large amount of training data is required, and when the similarity between the training data and the application environment is low, they cannot achieve good results. For example, using the training data of the indoor environment to model the outdoor environment is not effective. Moral et al. [22] proposed a place recognition algorithm, which was based on plane-based maps. However, their method could only be applied to indoor environments.

Segment-based place recognition in 3D point clouds (SegMatch) was presented in [2]. SegMatch first proposed a method based on segments by using a deep learning method. Random forest was used to match continuous segments. They firstly performed geometric verification tests on segment descriptors, which were fed to the recognition model. SegMatch extracts features, such as vehicles, trees, and buildings, so it could be used in both indoor and outdoor environments. However, SegMatch relies on the initial pose and does not work without a Global Positioning System (GPS) sensor. SegMatch’s construction of maps is less accurate and could only be used for map recognition.

At present, many slam methods, including closed-loop detection, have also appeared. [23] proposed a lightweight and ground-optimized slam method. It can complete the mapping work well, but its closed-loop detection sometimes has errors or missed recognition. [24] used the implicit moving least-squares (IMLS) surface to represent the model, thereby increasing the robustness of the system. At the same time, in addition to the above several traditional slam methods, the odometry estimation method using deep learning [25,26] has also been studied by many researchers but has not performed on par yet. Recently, Behley and Stachniss proposed a surfel-based motion estimation and mapping method, called SuMa [27]. SuMa allows us to represent large-scale environments and also maintains detailed geometric information of point clouds. Sparse point clouds are a challenge for it.

## 3. Proposed Methods

### 3.1. System Overview

**Figure 1:**
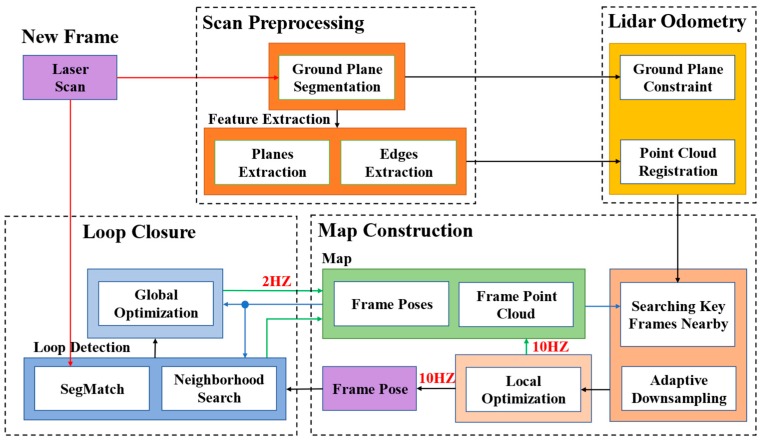
The system framework of our proposed optimized LOAM (lidar odometry and mapping) using ground plane constraints and SegMatch-based loop detection. The red arrows represent the input, the black arrows represent the process of the data transmission, the green arrows represent map generation, and the blue arrows represent the map call.

An overview of the proposed framework, which only received data from the lidar sensor, is shown in Figure 1. The system was mainly composed of four modules: scan preprocessing, lidar odometry, map construction, and loop closure. The first module mainly reduced noise, segmented the ground point cloud, and extracted feature point cloud from the original point cloud data. Under the constraints of the ground plane, the second module calculated the relative pose transformation between two consecutive frames roughly and quickly. The third module optimized the current pose based on the built map and added the current frame to the map. The entire map consisted of frames’ poses and point clouds. In addition, the optimized pose was transmitted to the fourth part. The loop detection in the last module was mainly composed of two parts, one was the neighborhood search detection, which only needed the pose data, and the other was the SegMatch detection, which needed the frame pose and the lidar point cloud. After detecting the loop, the module optimized the entire map. The system generated maps in real-time at a high frequency of 10 Hz and optimized the whole of the map at a low frequency of 2 Hz. Compared with the original LOAM framework of [1] and [8], our method paid more attention to the performance of ground vehicles. The details of each module are shown below.

### 3.2. Scan Preprocessing

**Figure 2:**
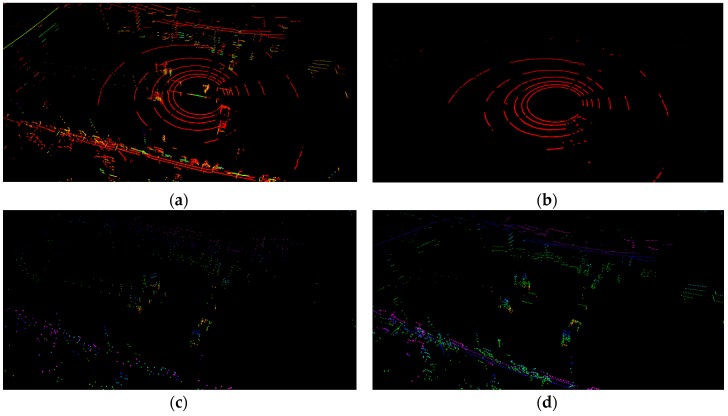
The original point cloud is shown in (**a**). The red points in (**b**) are ground plane points. Points in (**c**) and (**d**) are edge points and plane points, respectively.

The module mainly consisted of two parts, one was ground plane segmentation, and the other was feature extraction. The module mainly preprocessed the acquired original lidar point cloud. Firstly, during the robot’s movement, the data obtained by the laser would cause some distortion. Because we only had a lidar sensor, we assumed that the robot was moving at a constant speed, and the relative motion of two consecutive frames was similar. We directly used the relative motion of the previous frame to compensate for the current frame to remove distortion. What’s more, noise reduction was performed on the point cloud to eliminate individual outliers $F_{o}^{t}$. Then, the filtered point cloud $F_{f}^{t}$ was segmented to extract the ground plane point cloud. At last, feature extraction was performed after the ground plane was removed. The ground plane point cloud $F_{g}^{t}$, the extracted plane features $F_{p}^{t},$ and the edge features $F_{e}^{t}$ were transmitted to the next module. A visualization of a point cloud before and after ground plane segmentation is shown in Figure 2a–d.

#### 3.2.1. Ground Plane Segmentation

The ground plane point cloud is a good constraint on the pose estimation of the ground vehicle. For ground vehicles, ground point clouds tend to occupy one-third of the point cloud. Splitting out the ground plane point cloud greatly reduces the computation time of the later feature extraction. How to spend the shortest time to complete the ground plane segmentation is a key issue. This paper chose a random sample consensus (RANSAC) [3] to solve the above problem. According to the basic principle of RANSAC, three points are selected from each frame of the point cloud to obtain a plane. The commonly used plane equation is: ax+by+cz=d, where $a^{2}+b^{2}+c^{2}=1,d>0,\left(a,b,c\right)$ is the plane normal vector, and d is the distance from the lidar sensor to the plane. The specific steps are as follows:

1. After the noise reduction processing, randomly select three points $P_{1}\left(x_{1},y_{1},z_{1}\right)$, $P_{2}\left(x_{2},y_{2},z_{2}\right)$, $P_{3}\left(x_{3},y_{3},z_{3}\right)$ in the point cloud data P.
2. The plane S is determined according to three points $P_{1}\left(x_{1},y_{1},z_{1}\right)$, $P_{2}\left(x_{2},y_{2},z_{2}\right)$, $P_{3}\left(x_{3},y_{3},z_{3}\right)$. The values of the a, b, c, d parameters are determined by Equation (1).
  (1) $$\{\begin{array}{c} ax_{1}+by_{1}+cz_{1}=d\\ ax_{2}+by_{2}+cz_{2}=d\\ ax_{3}+by_{3}+cz_{3}=d \end{array}$$
3. Count the number of points on the plane S in P. Set the plane thickness ε (point to plane distance threshold) and calculate the distance di from any point $P_{i}\left(x_{i},y_{i},z_{i}\right)$ in P to plane S, where di is calculated by Equation (2):
  (2) $$d_{i}=\left|ax_{i}+by_{i}+cz_{i}-d\right|.$$
  Then, count the number of points of $d_{i}<\varepsilon$, and record it as the score of the plane S.
4. Repeat the above three steps K times and select the plane $S_{x}$ with the highest score.
  (3) $$1-1-C_{m}^{n}C_{m-1}^{n-1}C_{m-2}^{n-2}{}^{K}=\varphi$$
  In Equation (3), m is the number of points in the point cloud P, n is the number of points on the plane S, and φ is the probability that the ground plane is selected after K times of sampling. Since both m and n are large, we used approximate calculations here, and the simplified formula is as follows:
  (4) $$1-1-1-\tau^{3}{}^{K}=\varphi .$$
  In Equation (4), τ is the probability that the point is outside the plane $S_{x}$, and after simplification, K is obtained, as shown in Equation (5):
  (5) $$K=\frac{\log\left(1-\varphi \right)}{\log1-1-\tau^{3}}.$$
5. Re-fitting the selected ground plane data to obtain a ground plane parameter with less error.

According to the above steps, the ground plane point cloud can be extracted, as shown in Figure 2b. In this process, we first used a pass-through filter to extract point clouds ranging from 1.5 m to 2 m below the laser to avoid extracting the wall surface. We set the distance threshold to 0.2 and the max iteration number to 100. The ground plane point cloud was transmitted directly to the ground plane constraints of the next module. Feature extraction was performed on the remaining point clouds that did not contain the ground plane for later point cloud matching.

#### 3.2.2. Feature Extraction

The feature extraction module was similar to the method in LOAM [1]. Let *S* be the set of consecutive points of $p_{i}$ in a certain line of the lidar. $p_{i}$ is in the middle of *S*. We could calculate the curvature of the $p_{i}$ point in *S* according to the Equation (6).

(6) $$c=\frac{1}{\left|S\right|\cdot \left|\left|r_{i}\right|\right|}\left|\left|\underset{j\epsilon S,j\neq i}{\sum }\left(r_{j}-r_{i}\right)\right|\right|$$

Features were extracted using a method of calculating the curvature of each point. A point with a large curvature represented an edge feature, and a point with a small curvature represented a planar feature. Edge points and plane points are, respectively, shown in Figure 2c,d. However, unlike LOAM using all raw points, we extracted features from segmented points, which did not contain ground plane points. The number of segmented points occupied only two-thirds of the original point cloud data, which greatly reduced the computation time for feature extraction. The planar features and edge features extracted from each frame segmentation point cloud were transmitted to the next module.

### 3.3. Lidar Odometry

After obtaining ground plane points, edge points, and plane points, the module was committed to roughly performing pose estimation based on two consecutive frames of point clouds. In the LOAM’s method, the edge points $F_{e}^{t}$ and the plane points $F_{p}^{t}$ of the current frame were used to perform point-to-edge and point-to-plane scan-matching with the points $F_{e}^{t-1}$ and $F_{p}^{t-1}$ of the previous frame. However, for ground vehicles, if the above constraints were only used for matching, a serious matching error would generate, so we added a ground plane constraint to the matching constraints to reduce the error. The ground plane had a good constraint on $\left[t_{z},\theta_{roll},\theta_{pitch}\right]$, but had no constraint on $\left[t_{x},t_{y},\theta_{yaw}\right]$. Let the plane equation of the previous ground plane point cloud be ax+by+cz=d, where $a^{2}+b^{2}+c^{2}=1,d>0,\left(a,b,c\right)$ is the plane normal vector. The distance from the point $p_{g_{i}}\left(x_{g_{i}},y_{g_{i}},z_{g_{i}}\right)$ in the current ground plane point cloud $F_{g}^{t}$ to the plane was $d_{i}$. In order to ensure the robustness of the system, we did not directly use the plane parameters provided by RANSAC. Then, the following cost function was minimized to obtain the optimal solution:

(7) $$\min\left\{\underset{p_{i}\epsilon F_{g}^{t}}{\sum }{\left(ax_{g_{i}}+by_{g_{i}}+cz_{g_{i}}-d\right)}^{2}\right\}.$$

We solved the 6DOF pose $\left[t_{x},t_{y},t_{z},\theta_{roll},\theta_{pitch},\theta_{yaw}\right]$ of the robot based on matching the point cloud of frame t and frame t−1. $\left[t_{z},\theta_{roll},\theta_{pitch}\right]$ were mainly decided by ground plane constraints, and the remaining $\left[t_{x},t_{y},\theta_{yaw}\right]$ were mainly decided by the distance of point-to-edge and point-to-plane. If ground plane constraints were lost, $\;\left[t_{x},t_{y},t_{z},\theta_{roll},\theta_{pitch},\theta_{yaw}\right]$ were all determined by the distance of point-to-edge and point-to-plane. We used the least-square solver method in Ceres Solver [28] to solve poses. Compared with LOAM, we had a more efficient search method, so we reduced a lot of calculation time.

### 3.4. Map Construction

The pose error estimated by two consecutive frames was large, so we used the built map to optimize the current pose. This module *Map Construction* matched the edge point $F_{e}^{t}$ and the plane point $F_{p}^{t}$ with the features in the local map $M^{t-1}$ on the basis of the ground plane constraints and used the adaptive down-sampling method to improve the optimization efficiency. If we would have ignored the previous module *Lidar Odometry* and used this method directly for pose optimization, we would have spent a lot of time, and the system could not be real-time, so we used the frame pose $T_{L}^{t}$ estimated by the previous module *Lidar Odometry* as the initial value and combined the pose transformation $T_{M}^{t-1}$ to get the pose $T_{M}^{t}$ in the world coordinate system. The conversion relationship is as shown in Equation (8).

(8) $$T_{M}^{t}=T_{M}^{t-1}T_{L}^{t}$$

**Figure 3:**
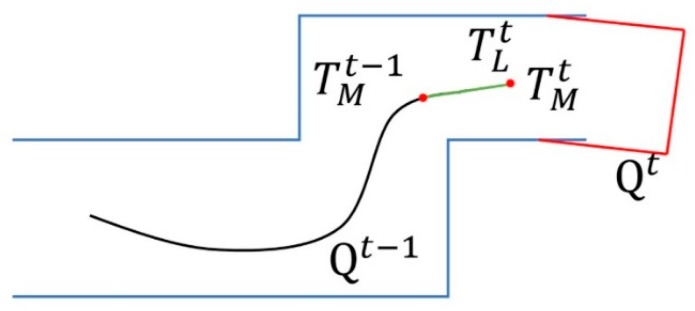
Illustration of point cloud map construction.

Finally, after the frame point cloud $F^{t}$ and the pose $T_{M}^{t}$ were associated, the point cloud was converted into $Q^{t}$ in the world coordinate system, as shown in Figure 3.

In order to facilitate the global optimization of loop detection, we recorded the feature points $F^{t}$ and corresponding pose $T_{M}^{t}$ of each frame to form a global map $N^{t-1}$, instead of using the method of saving all point clouds into a cube in LOAM [1]. $N^{t-1}$ is as shown in Equation (9).

(9) $$N^{t-1}=\left\{F_{e}^{1},\ldots ,F_{e}^{t-1},F_{p}^{1},\ldots ,F_{p}^{t-1},F_{g}^{1},\ldots ,F_{g}^{t-1},T_{M}^{1},\ldots ,T_{M}^{t-1}\right\}$$

This was somewhat similar to the method of LeGO-LOAM [23], but this paper also added the ground plane feature point $F_{g}$ to the global map $N^{t-1}$. We could construct the local map $M^{t-1}$ by using all the feature points in a certain range near the current feature point pose, but when there were more feature points in the range, the optimization time was increased. The pose estimated by the second module *Lidar Odometry* was not very accurate, so we used the third module *Map Construction* to optimize it. Under normal circumstances, the optimization took more time, so the third module *Map Construction* could not complete real-time optimization; thus, we used adaptive down-sampling to improve the real-time performance of the third module *Map Construction*. We had two ways to do adaptive down-sampling. The first method was to sample according to the distance from the point to the lidar. The laser was a divergent device. The closer it was to the lidar, the denser was the point cloud, so we divided the point cloud into three parts according to the distance. The closer the feature point cloud found to each frame was to the lidar, the fewer points were collected. The second way was to automatically adjust the search range based on the number of key points. At the same time, in order to ensure real-time optimization, we also performed additional down-sampling on the ground plane point cloud $F_{g}$.

After finding a suitable point cloud to form a local map $M^{t-1}$, the pose $T_{M}^{t}$ should be optimized so that the point cloud $Q^{t}$ could be well-matched with the local map $M^{t-1}$. Unlike LOAM [1], we still used the Ceres Solver [28] method to solve the problem to optimize the pose. Similarly, we added ground plane constraints to the optimization to obtain a more accurate pose estimation.

### 3.5. Loop Closure

The module consisted mainly of two parts. One was loopback detection, and the other was global optimization. When the system ran, it inevitably accumulated errors, so it could not constitute a more accurate global map. Thus, we added closed-loop detection. When this frame and the historical frame coincided, we optimized the global pose.

#### 3.5.1. Loop Detection

**Figure 4:**
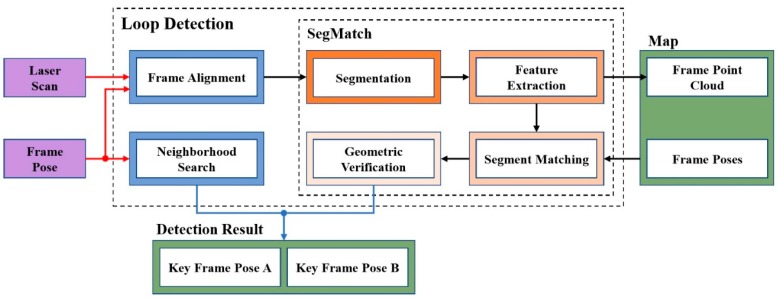
The overview of loop detection. The red arrow represents the input, the blue arrow represents the output, and the black arrow represents the transmission of the data.

After estimating the real-time pose of each frame, we needed to perform loop detection. The overview of loop detection is shown in Figure 4. Loop detection consisted of two parts, one was SegMatch, and the other was neighborhood search. Neighborhood search was only used to assist SegMatch for closed-loop detection only when SegMatch missed detection. The input was the pose $T_{M}^{t}$ of the current frame and the laser point cloud raw data. The output was the pose $T_{M}^{t_{A}}$ of the current keyframe and the pose $T_{M}^{t_{B}}$ of the historical keyframe with a high matching degree. At the same time, we saved the feature point cloud extracted in each frame into the map. The neighborhood search only needed the pose data of the current frame, and SegMatch needed not only the pose data but also the original laser point cloud data of the current frame. When we received the pose $T_{M}^{t}$ of the frame, the latest laser point cloud might be $L^{t+3};$ thus, the pose $T_{M}^{t}$ of the frame lagged behind the latest original laser point cloud data $L^{t+3}$. But SegMatch needed the frame pose $T_{M}^{t}$ and the laser point cloud $L^{t}$, so we had frame alignment of the two data. We kept the latest ten frames of laser point cloud data in a buffer {$L^{t-9},\ldots ,L^{t}$}. When we got the pose $T_{M}^{t}$ of a new frame, we looked for the corresponding laser point cloud data $L^{t}$ in the buffer based on the timestamp information. 

Since the pose of each frame was stored, the structure of the KD Tree could be used to manage the pose set $T_{M}$, which could greatly improve the search efficiency. Then, the historical pose $T_{M}^{i}$ in a certain range near the current pose $T_{M}^{t}$ was searched, and the point cloud of current pose $T_{M}^{t}$ was matched with the point cloud of pose $T_{M}^{i}$ using normal distribution transformation (NDT) [4] algorithm. If the score of matching was good enough, it was considered that the loop was detected. The pose $T_{M}^{i}$ with a short trajectory to the current pose $T_{M}^{t}$ was to be excluded. This was the neighborhood search method, and the SegMatch is as described below.

**Figure 5:**
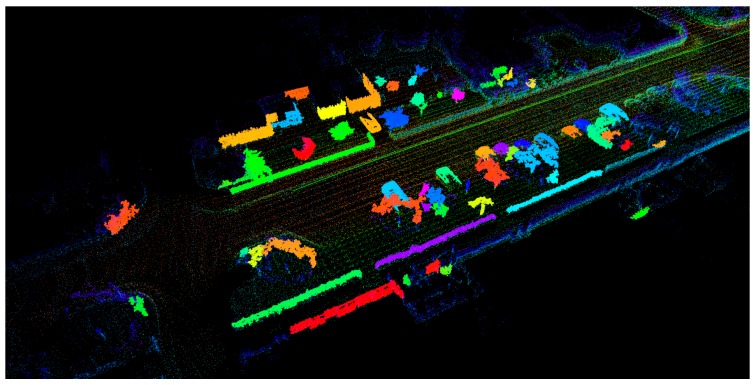
The global map, containing feature point cloud extracted by SegMatch.

SegMatch is a place recognition algorithm relying on matching 3D segmented point cloud. Based on the current frame pose $T_{M}^{t}$ and point cloud $L^{t}$, SegMatch could perform segmentation matching and loop detection. Unlike SegMap [29], we directly used the pose that LOAM estimated without using iterative closest point (ICP) [30] algorithm for matching estimates. We didn’t use SegMatch to optimize the global pose but for the loop detection. The SegMatch algorithm is mainly divided into four steps:

1. Segmentation. After the pose $T_{M}^{t}$ was associated with the point cloud $L^{t}$, the local point cloud was extracted in the neighborhood of the current pose $T_{M}^{t}$. The extracted point cloud was filtered using a voxel grid, and then the filtered point cloud was segmented into a set of point clusters $C^{t}$ using the “Cluster-All Method” of [31].
2. Feature Extraction. Feature extraction was performed on the segmented cluster $C^{t}$ using several different descriptors. The descriptors used in this paper were calculated based on the feature vector $f^{t}=\left[f_{1}^{i}f_{2}^{i}\ldots f_{m}^{i}\right]$. One of the descriptors contained seven features, as proposed in [32]: linearity, planarity, scattering, omnivariance, anisotropy, eigenentropy, and change of curvature. We stored the feature point cloud $F_{s}^{t}$ extracted every frame into the map $N^{t-1}$ for global recognition later, as shown in Figure 5.
3. Segment Matching. The extracted point cluster $C^{t}$ of the current frame was matched with the extracted set of point clusters $\left\{C^{1},\ldots ,C^{t}\right\}$ in the global map $N^{t-1}$. The point cluster $C^{i}$ in the set of point clusters $\left\{C^{1},\ldots ,C^{t}\right\}$ was associated with the pose $T_{M}^{i}$, and the pose $T_{M}^{i}$ was updated in real-time. To determine whether there was a match between the current frame and the historical frame, we chose the deep learning method. In order for the random forest classifier to identify if the two clusters were matched, we calculated the absolute difference between the two eigenvectors:
  (10) $$\Delta f_{1}=\left|f_{1}^{i}-f_{1}^{j}\right|.$$
4. Geometric Verification. Finally, the geometric consistency of the segment cluster $C^{t}$ was determined using random sample consensus (RANSAC) [3], so that the pose $T_{M}^{t}$ of the current frame and the pose $T_{M}^{x}$ of the history frame satisfying the condition were obtained.

#### 3.5.2. Global Optimization

When two keyframe poses were detected, we used the GTSAM algorithm [33,34] to optimize the global pose. The factor graph GTSAM used connects poses using factors, such as constraints and measurements. GTSAM used a nonlinear optimizer to solve the factor graph to determine the most likely configuration of the current and past poses. Each time we estimated a new pose, we used the algorithm framework to establish the constraint between the current frame pose $T_{M}^{t}$ and the previous pose $T_{M}^{t-1}$. After receiving the two frame poses $T_{M}^{t}$ and $T_{M}^{x}$ from the loop detection, we then established a constraint between the two in the algorithm framework to complete the global pose optimization. In this way, assuming the noise model of each sensor and actuator is correct, accurate robot trajectories and environmental maps could be determined.

## 4. Experiment

We did a series of experiments to evaluate the proposed method. This chapter is divided into two parts. First, we tested our algorithm on different data sets and showed the results. Second, we had separately analyzed the improved modules and the operating speed of the system.

### 4.1. Tests

In this section, we tested with a dataset only containing laser data and compared the results to other lidar-based SLAM algorithms. We tested our algorithms using the data from HDL-64E and VLP-16, respectively. In detail, we used the KITTI dataset [10], which was captured by an HDL-64E in an urban environment, to test our method. In addition, we used our own experimental platform, which used VLP-16 to sense the environment, to record indoor and outdoor sequences, and tested them using the proposed method. Our proposed algorithms ran on a laptop computer with 2.2 GHz quad cores and 6Gib memory in Ubuntu16.04 based on the robot operating system. We used the voxelized grid approach with a leaf size 0.1 to down-sample the edge point cloud, with a leaf size 0.2 to down-sample the plane cloud, and with a leaf size 0.4 to down-sample the ground point cloud for each laser scan. In order to get more convincing results, we did each experiment five times and calculated their average.

#### 4.1.1. Tests with KITTI Dataset

**Figure 6:**
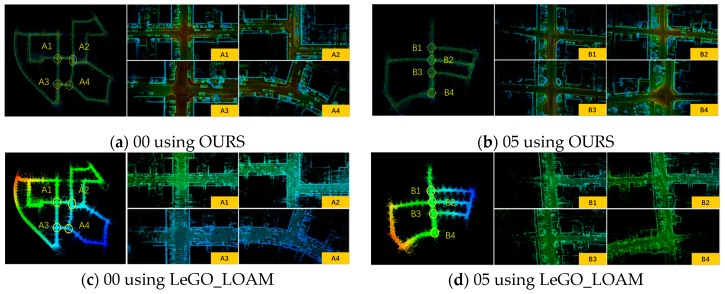
The result of our algorithm on the KITTI dataset. (**a**) is sequence 00, and (**b**) is sequence 05 using our proposed method. (**c**) is sequence 00, and (**d**) is sequence 05 using LeGO_LOAM. A1–A4 and B1–B4 are all the loop closure areas.

We tested our algorithm on the public dataset KITTI. More specifically, we used the sequences “00” and “05” that contained the most loops where the vehicle revisited the same environment. Sequence 00 lasted 3.7 km, and sequence 05 lasted 2.2 km in an urban environment. We mainly validated the closed-loop performance of our algorithm using the public dataset. The test of our method over the datasets ran at real-time speed and only used the lidar of Velodyne HDL64. At the same time, we have also shown the results of LeGO_LOAM to compare with our method. The experimental results are shown in Figure 6.

Both of the sequences were long-distance datasets. The estimation errors inevitably occurred with time, but we could see from Figure 6 that our algorithm could construct a point cloud map with global consistency, and the optimization task could be completed well in the revisited areas. It could be seen that our method was similar to LeGO_LOAM. In order to ensure the real-time performance of the system, we used a voxel grid approach with a leaf size 0.4 to down-sample the point cloud. Finally, our algorithm only lost about 50 frames and constructed a dense point cloud map. LeGO_LOAM’s optimization frequency was relatively low, only one-fifth of the keyframes were retained, so the map was relatively sparse. Figure 6c,d is overlapping and fuzzy, and the details could not be clearly seen. The areas of A1–A4 and B1–B4 in Figure 6 were the loop closure areas, where the vehicle passed at least twice. It could be seen that there was only a small drift in these places. 

**Figure 7:**
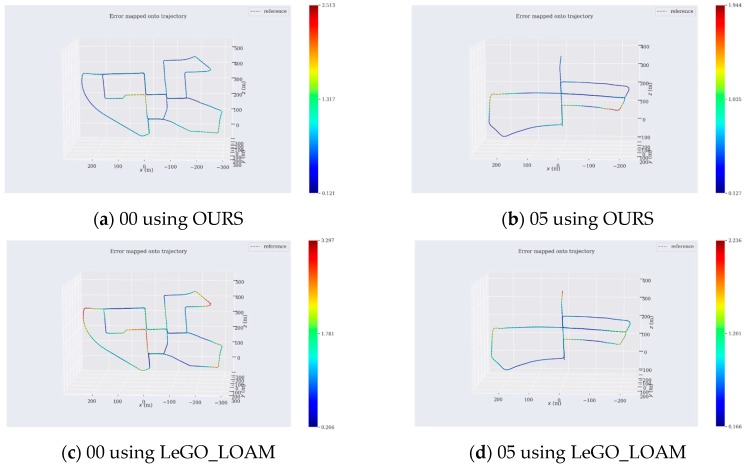
Error mapped onto trajectory. (**a**) is sequence 00, and (**b**) is sequence 05 using our method. (**c**) is sequence 00, and (**d**) is sequence 05 using LeGO_LOAM. The dotted trajectory is ground truth, and the colored trajectory is the trajectory using our method and LeGO_LOAM. The error is as shown on the right ruler. Blue represents the smallest error, and red represents the largest error. The coordinate system in the figure is the same as in LOAM. The z-axis is facing forward, the x-axis is facing left, and the y-axis is facing upward.

To more intuitively analyze the accuracy of the map we built, we compared the trajectory that we drew with the provided ground truth. In addition, we compared the trajectory drawn by LeGO_LOAM with the ground truth to draw an error trajectory map. The results are shown in Figure 7. It could be seen from Figure 7a,b that the closed-loop optimized trajectory using our method could well fit the ground truth. Most of the color of the trajectory was blue, indicating that the trajectory error estimated using our method was small. There were a few errors in the corners, which should be due to the fact that the car was turning too fast and causing a certain distortion. It was known from Figure 7c,d that although LeGO_LOAM could realize closed-loop detection, the error trajectory drawn by Lego_loam had fewer blue areas, indicating that the error was generally larger. In order to more intuitively see the error size of each figure, we calculated and listed the corresponding errors, as shown in Table 1. It could be seen from Table 1 that the mean value error and the mean square error of the map optimized by loop closure using our method were within 1 m in the range of up to kilometer, and our error results were better than LeGO_LOAM’s, so that the superiority and robustness of our algorithm could be seen in the long-distance outdoor environment.

#### 4.1.2. Tests with Our Dataset

**Figure 8:**
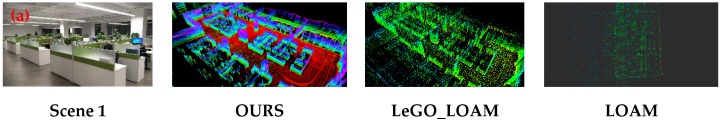

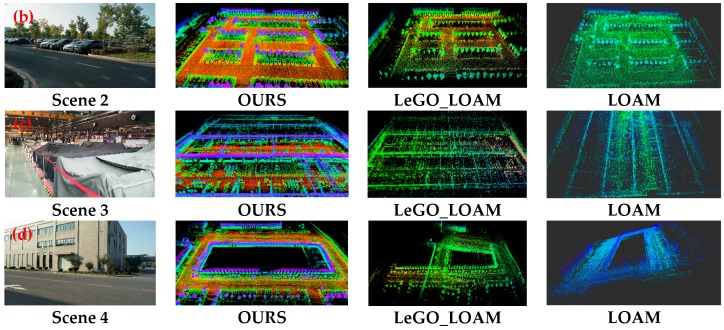
(**a**) shows a point cloud map of an indoor office built using three methods. (**b**) shows a three-dimensional point cloud map of the parking lot constructed by three different methods. (**c**) shows the point cloud map of the complex environment in a large workshop constructed by three algorithms. (**d**) shows the point cloud map of the road scene around the building constructed by three SLAM (simultaneous localization and mapping) methods.

To test the robustness of the algorithm, we tested the indoor and outdoor environments separately. The first scene was an office covering 120 square meters on the third floor of the “C1” building in SIASUN. The second scene was the parking lot between the “C1” building and the “C5” building in SIASUN, which was 80 m long and wide. The third scene was a workshop with a length and width of 100 m in the “C2” building of SIASUN, but we only walked two of the aisles. The last scene was the road around the “C5” building in SIASUN, which was 100 m long and 50 m wide. Our experimental results were compared and analyzed with LOAM and LeGO_LOAM. The test results are shown in Figure 8a–d.

According to the point cloud diagram of Figure 8a, since the test environment was relatively small and regular, the three methods could complete the map construction well, and the error was small. However, as the scene size increased, the error gradually increased, as shown in Figure 8b,c. The three methods could basically construct the maps of Scene 2 and Scene 3, but in contrast, the map constructed by our method was more dense and complete. LOAM and LeGO_LOAM relied on the Inertial Measurement Unit (IMU) to obtain a more accurate initial pose, but in this paper, we only used the lidar sensor, so the calculation error of these two methods gradually increased with time. LeGO_LOAM had loop detection, so the cumulative error could be reduced after the loop was detected. In the last scene, only our method could build a complete map, as shown in Figure 8d. LOAM had no loop detection, so the z-axis drift could not be corrected, resulting in a large deviation. Since the road between two buildings was similar to the long corridor scene, LeGO_LOAM could not overcome this difficulty, so the map construction failed.

**Figure 9:**
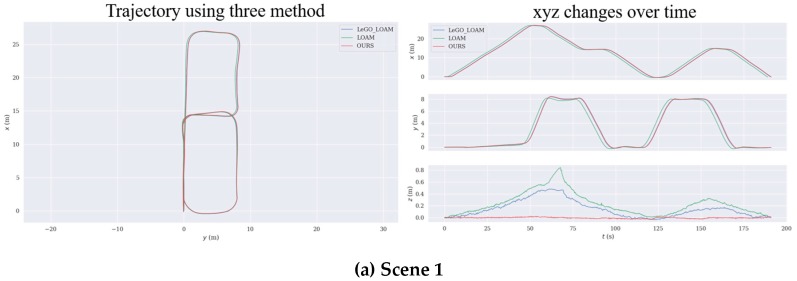

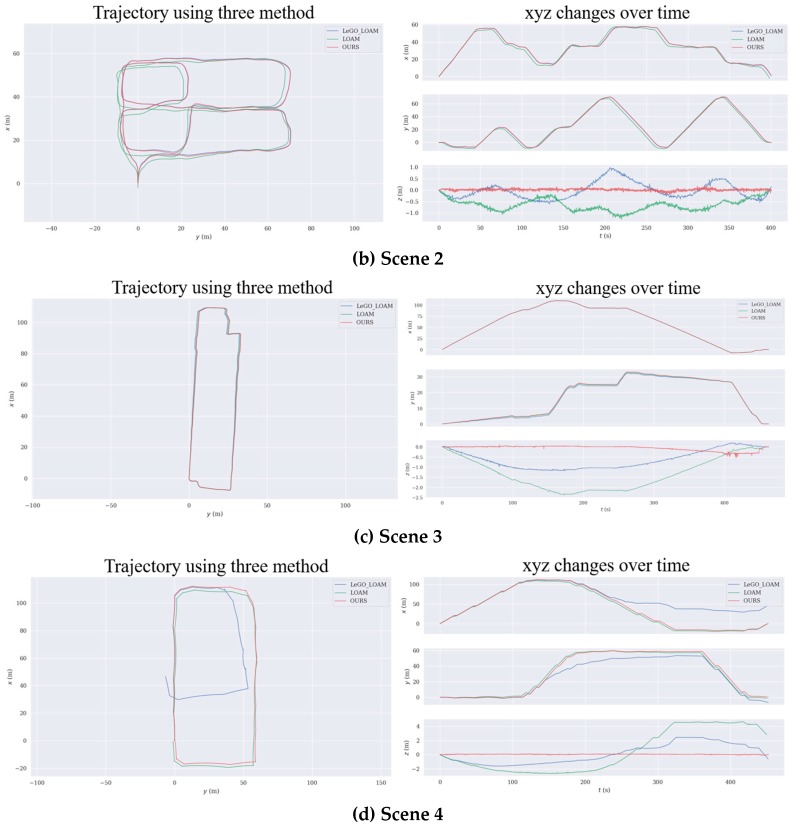
(**a**) shows the trajectory of the office environment estimated using the three algorithms. (**b**) shows the trajectory estimated in the parking lot using three methods. (**c**) shows the trajectory in the workshop estimated by three different methods. (**d**) shows the trajectory of the road around a building estimated by the three SLAM algorithms. In the four figures, the blue line is LeGO_LOAM, the green line is LOAM, and the red one is OURS. The plot uses a common coordinate system. The x-axis is the robot’s forward direction, the y-axis is the left side of the robot, and the z-axis is upward. The starting points in the figure all start from the origin (0,0). The left half of each scene is a top view of the trajectory, and the right half is the changes of xyz over time.

During the experiment, we preserved their real-time estimated poses while using three algorithms to construct four different scene maps. The trajectories estimated by the three algorithms in the four scenes are shown in Figure 9a–d.

Due to experimental conditions, we were unable to obtain accurate trajectories. We started from the marked starting point, and finally returned to the starting point, and compared the final deviation. For Scene 1 and Scene 3, the trajectories estimated by the three methods were similar, but only our method and LeGO_LOAM implemented closed-loop detection to optimize the global pose. LOAM had the worst ground constraints effect, and our ground constraints effect was the best. For Scene 2, the maps constructed by the three methods had good global consistency, but the error of the LOAM method was larger than the other two methods. Our ground constraint was better than the other two methods. For Scene 4, from Figure 9d, we could clearly see that the LeGO_LOAM trajectory deviated from the correct track due to the wrong loop detection, and LOAM did not achieve the re-identification of the map at the end. Only our method could complete the map construction task well.

In order to verify the global consistency of the map, we set the initial pose of each test to [0,0,0,0,0,0] and compared the relative pose estimation error between the final pose and the initial pose. The results are shown in Table 2.

It could be seen from Table 2 that the consistency of Scene 1 and Scene 3 was better, and the errors were within 1 m. LeGO_LOAM had ground constraints and closed-loop detection, so the final pose deviation was not great, especially the z-axis deviation, but the z-axis deviation of LOAM was large. In Scene 2, only the error of our method was within 1 m, and the other two were relatively large. The final pose estimated using LeGO_LOAM had a large deviation on the z-axis, but it could be seen from Figure 9b that the average z-axis deviation of LeGO_LOAM was less than LOAM. In Scene 4, the consistency of LOAM was also poor, and the z-axis deviation was very large. Our method used ground plane constraints and SegMatch-based loop detection to construct the above four scenes maps well.

### 4.2. Discussion of Tests

In this section, in order to further analyze the advantages and performance of our proposed algorithm, we tested and analyzed the ground plane constraints, loop closure, and runtime.

#### 4.2.1. Influence of Ground Plane Constraints

**Figure 10:**
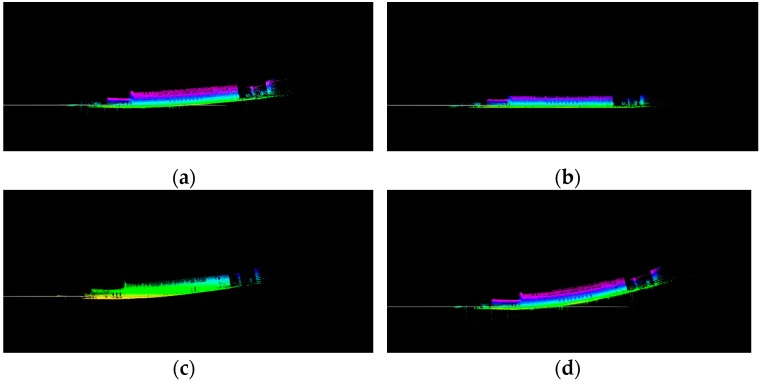
(**a**) shows the effect of our algorithm without ground plane constraints and with a high frequency of 10 Hz to optimize map, and (**b**) shows the effect of our algorithm with ground plane constraints. (**c**) shows the test results of the LeGO_LOAM algorithm. (**d**) shows the effect of our method without ground plane constraints and with a low frequency of 2 Hz to optimize the map.

To test the performance of the ground plane constraints, we recorded a short dataset that was straight 100 m on the flat ground. The result was obtained by observing the deviation of the z-axis, and the test results are shown in Figure 10a–d.

Figure 10a is a map constructed using our SLAM algorithm without ground plane constraints, and it was clear that the z-axis drift was getting larger and larger. The accumulated errors had a huge impact on the later construction, which was unfavorable to this system. After adding the ground plane constraints, it could be clearly seen that the z-axis drift was controlled, as shown in Figure 10b. The ground plane constraints were also included in LeGO_LOAM, so we also tested the dataset using this algorithm. The experimental results are shown in Figure 10c. It could be seen from Figure 10c that even if there were ground plane constraints in LeGO_LOAM, the z-axis drift still existed. In addition, we also found that our method without ground plane constraints and with a high frequency of 10 Hz to optimize the map was better than LeGO_LOAM. When we reduced the optimization frequency to the same as LeGO_LOAM, our ground constraints effect would be worse than LeGO_LOAM.

**Figure 11:**
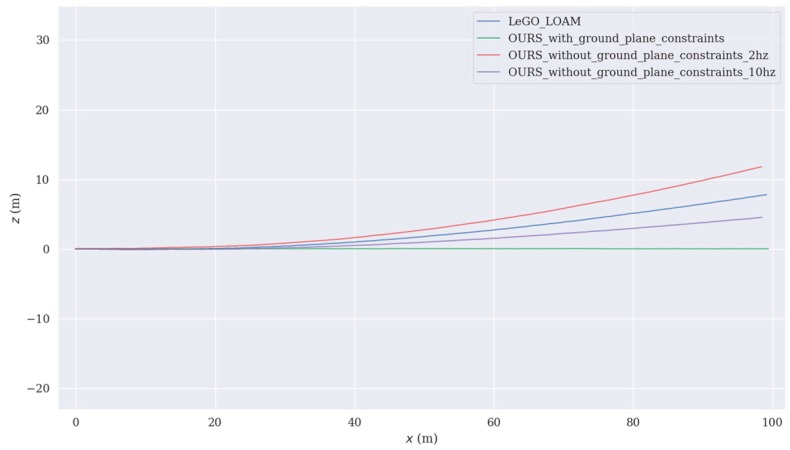
The trajectory of four methods. The red line is the trajectory estimated using our algorithm without ground plane constraints and with a low frequency of 2 Hz to optimize map, the blue line is the trajectory estimated using LeGO_LOAM, the purple line is the trajectory estimated using our algorithm without ground plane constraints and with a high frequency of 10 Hz to optimize map, and the green line is the trajectory estimated using our algorithm containing ground plane constraints.

In order to more intuitively view the size of the drift, we plotted the trajectory of the three, as shown in Figure 11. In comparison, our ground plane constraints algorithm had obvious advantages and performance better than LeGO_LOAM.

#### 4.2.2. Impact of Loop Closure

**Figure 12:**
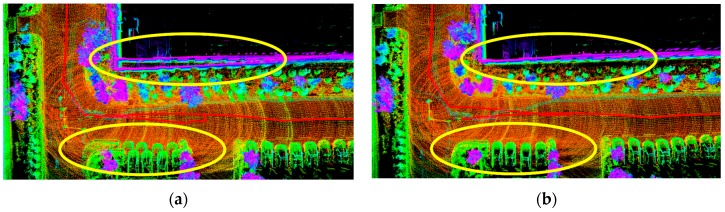
The effect of our loop closure algorithm. (**a**) Without loop closure, (**b**) with loop closure.

Loop detection could correct the established map, which was of great significance for pose estimation. From Section 4.1.1, a significant effect of closed-loop detection in long-distance urban environments could be seen. To test the performance of loop detection and optimization more specifically and intuitively, we recorded a circular trajectory dataset and tested the effects of the closed-loop and no-closed-loop algorithms, as shown in Figure 12. It could be clearly seen from Figure 12a that the car and the wall had a ghost image, which was the phenomenon that the map was not corrected. When the loop closure was added, this part was optimized, and the ghosting phenomenon disappeared, as shown in Figure 12b. Therefore, our SegMatch-based loop detection algorithm could eliminate the cumulative error well when the loop was detected.

#### 4.2.3. Runtime Analysis

In order to explore the real-time nature of the system, we analyzed the runtime of each module in our algorithm. The results are shown in Table 3. Each of the modules ran on a different thread. As could be seen from Table 3, the first three modules took less than 100 ms. Our adaptive down-sampling method guaranteed the real-time performance of the system, and a high-precision dense point cloud map could be constructed. The loop closure module took much time, and time would increase as the map got bigger, but did not affect the overall performance of the system, as it only needed to optimize the global map at a frequency of 2 Hz.

## 5. Conclusions

In this paper, we proposed optimized lidar odometry and mapping method using ground plane constraints and SegMatch-based loop closure. The use of ground plane constraints increased the accuracy of point cloud registration. The adaptive down-sampling method was used to improve the real-time performance of local optimization so that a dense point cloud map could be constructed. SegMatch was used to perform loop detection on the map built by LOAM to improve the global map.

The proposed method was firstly tested in four scenes of our own datasets and compared with the two existing methods. The results showed that our method could achieve low-drift real-time localization and mapping in large scenes. At the same time, we also tested on the public dataset KITTI for closed-loop detection and global optimization in the long-distance urban environment and construction of a 3D point cloud map. We evaluated the performance of the system from three aspects: ground plane constraints, loop closure, and runtime. The results showed the superiority of our algorithm.

In future work, we would complete the multi-sensor fusion based on IMU to improve the robustness and accuracy of the system. Secondly, we would study the filtering method of the original point cloud to quickly remove dynamic objects and increase the anti-interference ability of the system. We would broaden our application area to underground garage scenes to resolve an autonomous parking problem for unmanned vehicles.

## Figures and Tables

**Table 1:** Pose estimation error on on KITTI dataset.

Sequence	Method	Max (m)	Min (m)	Mean (m)	Rmse ^1^ (m)
00	OURS	2.51	0.12	0.75	0.82
	LeGO_LOAM	3.29	0.26	1.45	1.61
05	OURS	1.94	0.12	0.67	0.76
	LeGO_LOAM	2.24	0.17	0.83	0.89

^1^ Rmse: Root Mean Square Error.

**Table 2:** Relative pose estimation error when returning to start in four scenes using three different algorithms.

Scene	Method	Roll	Pitch	Yaw	Total Rot.^2^ (deg)	X	Y	Z	Total Trans.^3^ (m)
1	OURS	−0.52	0.48	−179.74	179.74	−0.08	−0.05	−0.01	0.08
	LOAM	−1.51	−0.33	−179.88	179.88	−0.18	−0.05	0.03	0.19
	LeGO_LOAM	−0.81	−0.4	−179.59	179.6	−0.12	−0.06	0.02	0.13
2	OURS	4.34	0.01	179.75	179.8	0.47	−0.19	0.01	0.51
	LOAM	2.71	0.8	174.69	174.72	−2.06	−0.02	0.03	2.06
	LeGO_LOAM	1.27	−0.72	177.93	177.93	1.22	−0.13	0.16	1.24
3	OURS	−1.49	0.44	0.94	1.83	−0.05	0.01	−0.01	0.04
	LOAM	0.43	−0.61	−6.4	6.44	−0.16	0.09	−0.09	0.21
	LeGO_LOAM	−0.83	0.87	0.3	1.24	−0.15	0.02	−0.01	0.15
4	OURS LOAM LeGO_LOAM	2.57 −3.31	−1.98 −1.58	−8.13 −9.52	8.76 10.2	−0.01 −0.51 fail	0.11 −1.19	0.04 2.82	0.11 3.11

^2^ Rot.: Rotation; ^3^ Trans.: Translation.

**Table 3:** Runtime performance for each module.

Modules	Average Time (ms)
Scan preprocessing	50
Lidar odometry	65
Point cloud map construction	80
Loop closure	220
